# CD82 palmitoylation site mutations at Cys5+Cys74 affect EGFR internalization and metabolism through recycling pathway: CD82 palmitoylation mutation can regulate the localization of EGFR

**DOI:** 10.3724/abbs.2022011

**Published:** 2022-02-23

**Authors:** Jingya Bu, Weiliang Zhong, Meixian Li, Shuiqing He, Mingzhe Zhang, Yu Zhang, Ying Li

**Affiliations:** 1 Department of Clinical Laboratory the Second Affiliated Hospital of Dalian Medical University Dalian 116023 China; 2 Department of Orthopaedics Surgery the First Affiliated Hospital of Dalian Medical University Dalian 116011 China; 3 Key Laboratory of Molecular Mechanism for Repair and Remodeling of Orthopaedic Diseases Liaoning Province Dalian 116011 China; 4 Department of Clinical Laboratory Jiangxi Maternal and Child Health Hospital Nanchang 330000 China.

**Keywords:** tetraspanin, CD82, palmitoylation, EGFR, Rab11a

## Abstract

Tetraspanin CD82 often participates in regulating the function of epidermal growth factor receptor (EGFR) and hepatocyte growth factor receptor (c-Met). Palmitoylation is a post-translational modification that contributes to tetraspanin web formation and affects tetraspanin-dependent cell signaling. However, the molecular mechanisms by which CD82 palmitoylation affects the localization and stability of EGFR and c-Met have not yet been elucidated. This study focuses on the expression and distribution of EGFR and c-Met in breast cancer as well as the related metabolic pathways and molecular mechanisms associated with different CD82 palmitoylation site mutations. The results show that CD82 with a palmitoylation mutation at Cys5+Cys74 can promote the internalization of EGFR. EGFR is internalized and strengthened by direct binding to CD82 with the tubulin assistance and located at the recycling endosome. After studying the recycling pathway marker proteins Rab11a and FIP2, we found that formation of the EGFR/CD82/Rab11a/FIP2 complex promotes the internalization and metabolism of EGFR through the recycling pathway and results in the re-expression of EGFR and CD82 on the cell membrane.

## Introduction

The tetraspanin CD82, belonging to the family of tetraspanins, is a small membrane protein with four transmembrane regions. CD82 is encoded by the *KAI1* gene, and as a recognized tumor suppressor factor, it is widely distributed in various normal tissues [ 1, 2]. In addition to four transmembrane regions, CD82 contains an extracellular small loop (EC1), an extracellular macroloop (EC2), and an intracellular small loop [ 3, 4]. In the variable region of EC2, there are sites that can bind to other proteins. This structural feature helps the formation of subsequent tetraspanin network. In the CD82 transmembrane domain, there are three highly conserved polar residues that can interact with the transmembrane domains of other tetraspanins, and therefore, CD82 can connect to other types of tetraspanins, forming compounds with specific functions [5]. In addition, CD82 can directly or indirectly bind to signal molecules, such as integrins, EGFR, c-Met and G protein-coupled receptors, forming microdomains enriched with tetraspanins on the cell membrane [ 6, 7].

EGFR is one of the main members of the ErbB family, which plays a regulatory role in embryonic development, tissue differentiation, and tumorigenesis and development [8]. Previous studies have shown that in solid tumors the overexpression of EGFR is typically related to the increase in the secretion of homologous ligands, which leads to the chronic activation of EGFR. When EGFR is not activated, CD82 can downregulate EGFR expression by regulating the internalization kinetics. CD82 can also cooperate with vesicle-associated membrane proteins and actin, thereby changing the signal transduction pathway of EGFR [ 9, 10]. c-Met is also a receptor tyrosine kinase, which is primarily expressed in epithelial and endothelial cells [11]. As the only high-affinity receptor for hepatocyte growth factor, c-Met exhibits a trend of overexpression in breast cancer, pancreatic cancer, gastric cancer, and other tumors. Both EGFR and c-Met are tumor metastasis-related receptors that are involved in the regulation of tumor cell metastasis, and have been extensively studied. CD82 can directly inhibit the c-Met expression and reduce cell invasion and growth by weakening the signal interaction between c-Met and integrin [ 12– 14].

Palmitoylation modification is an important and common post-translational modification process of proteins. At present, the most widely investigated process is S-palmitoylation, that is, the addition of a 16-carbon palmitate to the cysteine (Cys) residue of proteins. Lipid bonds on palmitoyl groups can bind to Cys residues and affect protein expression and function [ 15, 16]. The palmitoylation mutation of the tetraspanin protein can promote the formation of the tetraspanin protein network, which is conducive to the biological function of the tetraspanin protein-enriched microdomain. Although it has been established that the palmitoylation of CD82 can regulate the biological characteristics of tumor cells [17], its specific molecular mechanism remains to be studied. CD82 contains five cysteine residues in the proximal membrane region, i.e., Cys5, Cys74, Cys83, Cys251, and Cys253. The relationship between CD82 palmitoylation mutation and EGFR and c-Met has not been clarified so far, including the theoretical mechanism for its expression and localization changes in tumor cells and specific metabolic pathways. Therefore, in this study, we compared the distribution and metabolic pathways of EGFR and c-Met associated with different palmitoylation mutants of CD82 and explored the corresponding molecular mechanisms.

## Materials and Methods

### CD82 palmitoylation mutation plasmid construction

The flow chart of CD82 palmitoylation mutant plasmid construction was shown in Supplementary Figure S1A. On the basis of the wild-type CD82 plasmid, some of the cysteine residues of the CD82 palmitoylation site were mutated to alanine. According to the CDS sequence of CD82, the primers of the CD82 palmitoylation mutation were designed as shown in Supplementary Table S1. TransStart® FastPfu Fly DNA Polymerase (AP231-01; TransGen Biotech, Beijing, China) was used to run the PCR reactions. The correctness of CD82 palmitoylation site mutation plasmid was verified by sequencing (General Biol, Hefei, China).

### Experiment grouping

**Figure 1:**
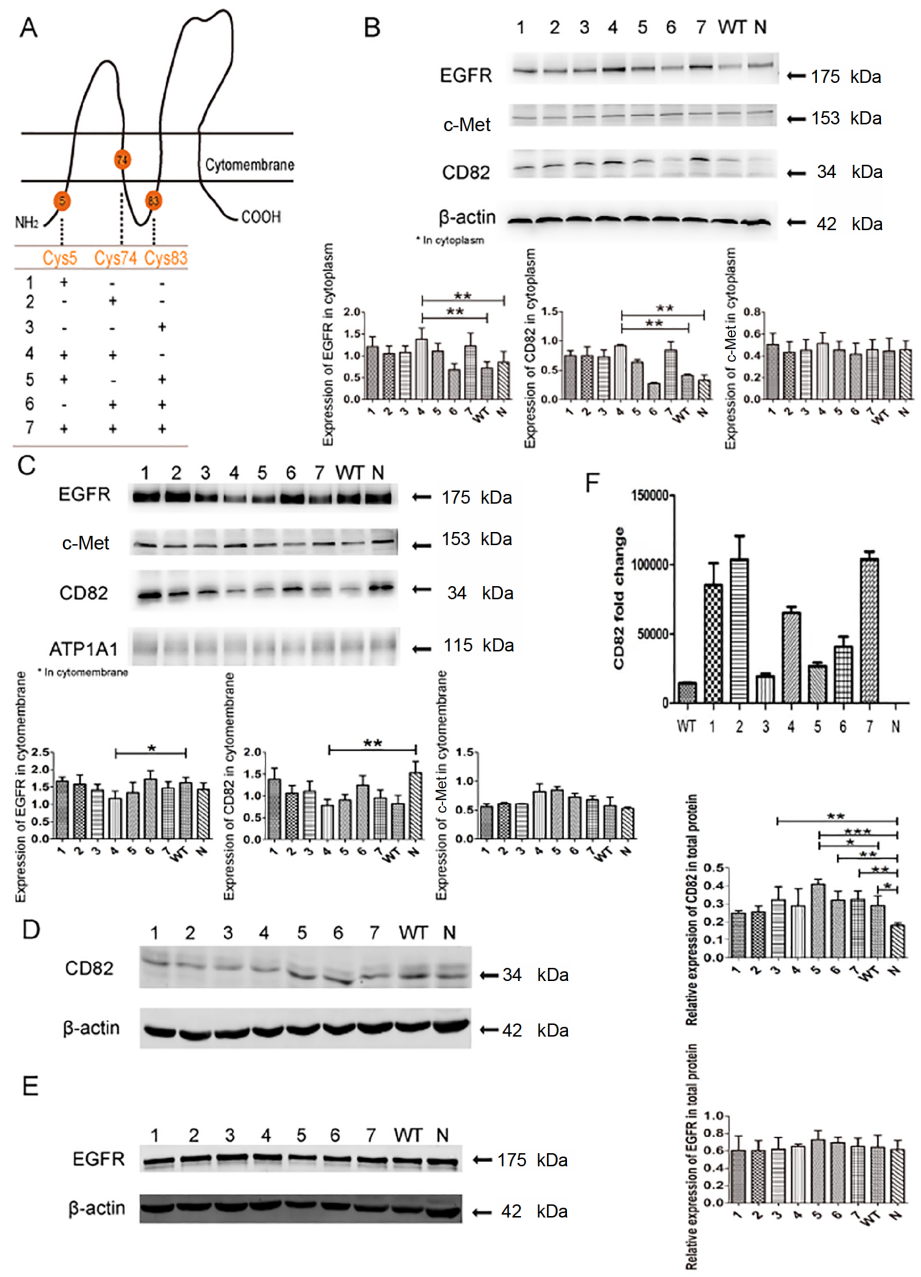
Figure1 **The expression and localization of EGFR and c-Met with different palmitoylation site mutations in CD82**(A) Schematic diagram of mutations at different palmitoylation sites of CD82. "+" represents mutation and "-" represents no mutation. (B) and (C) Western blot analysis results confirmed EGFR, CD82 and c-Met expressions in the cytoplasm and cytomembrane after mutations at different palmitoylation sites of CD82. (D) and (E) The expressions of CD82 and EGFR in the total protein after different mutations at palmitoylation sites of CD82. (F) The levels of CD82 after the transformation of different mutants of CD82 determined by qPCR.

Three sites, Cys5, Cys74, and Cys83, in the cytoplasmic proximal membrane area were selected for single-point (Cys5, Cys74, or Cys83), double-point (Cys5+Cys74, Cys5+Cys83, or Cys74+Cys83), and triple-point mutation (Cys5+Cys74+Cys83) analysis. The palmitoylation site mutations of CD82 are shown in Figure 1A. A certain number of cells were counted in each group to calculate the average fluorescence intensity.

### Cell culture and transfection

Breast cancer MDA-MB-231 cells (FuHeng Biology, Shanghai, China) were cultured in Dulbecco’s Modified Eagle Medium (DMEM) basic culture medium (Gibco, Carlsbad, USA) supplemented with 10% fetal bovine serum (FBS; AusGeneX, Brisbane, Australia) in 5% CO _2_. The cell lines used in our experiments were free of mycoplasma infections. HighGene transfection reagent (RM09014; Abclonal, Wuhan, China) was used for the transfection of MDA-MB-231 cells. The cells were inoculated into 6-well plates and considered ready for transfection when the cell density reached 70% to 90%. A total of 3 μg plasmid (General Biol) was added to 200 μL of serum-free opti-MEM medium (Gibco) and mixed well. Afterward, 6 μL of HighGene transfection reagent was added and mixed well. The mixture was evenly dripped into 6-well plates and mixed. After 4–6 h of transfection, the medium was replaced by complete medium with 10% FBS, and continued to cultivate for 24–48 h before the subsequent experiments.

### Protein extraction and western blot analysis

The Membrane and Cytosol Protein Extraction Kit (P0033; Beyotime, Shanghai, China) was used to cluster the cell total proteins into cytomembrane and cytoplasm proteins. Total protein was extracted using the whole protein extraction kit. Proteins were separated by 10% sodium dodecyl sulfate-polyacrylamide gel electrophoresis (SDS-PAGE) and transferred to PVDF membranes, which were then blocked in QuickBlock™ for 10 min and incubated with anti-CD82 antibody (ab66400; 1:1000; Abcam, Waltham, USA) overnight at 4°C. After being washed, the membranes were incubated with the DyLight® 680-Goat anti-Rabbit IgG (H+L) secondary antibody (35568, 1:10,000; Invitrogen, Carlsbad, USA) for 1 h at room temperature. The Odyssey fluorescence scanning imaging system (LI-COR, Lincoln, USA) was used for protein detection.

### Real-time quantitative PCR

RNA was extracted by using TaKaRa MiniBEST kit (Dalian, China). SYBR Green method was used to perform reverse transcription according to the manufacturer’s instructions. The mRNA level of GAPDH was used as an internal control. The primer sequences used were: *CD82* forward, 5′-GCCGACAAGAGCAGTTTCAT-3′, reverse, 5′-CAGCTTGCCCATGTTGAAGT-3′. *GAPDH* forward, 5′-CAAGCTCATTTCCTGGTATGAC-3′, reverse, 5′-CAGTGAGGGTCTCTCTCTTCCT-3′.

### Co-immunoprecipitation assay

The transfected MDA-MB-231 cells were collected and lysed with NP-40 Lysis Buffer (N8032; Solarbio, Beijing, China) supplemented with protease inhibitor (Beyotime) according to the manufacturer’s instructions. A total of 500 μg of cellular extract was incubated with mouse immunoglobin G (ABclonal) at 4°C overnight. Protein A+G Agarose beads (P2055; Beyotime) and appropriate anti-CD82 primary antibody (ab59509; Abcam) were used for co-immunoprecipitation overnight at 4°C. After being washed with PBS three times, the beads were heated at 100°C for 5 min so that the proteins were dissociated from the beads, and then centrifuged to collect the protein. Finally, the target protein was detected by western blot analysis.

### Immunofluorescence staining

Cells were fixed with 4% paraformaldehyde (PFA) for 15 min and with 1% NP-40 for 20 min at 25°C, and then washed with PBS three times. Afterward, 5% normal goat serum was used for blocking at 37°C for 30 min. The cells were incubated with rabbit anti-EGFR primary antibody (A11577, 1:100; ABclonal) overnight at 4°C, followed by incubation with fluorophore-conjugated secondary antibodies (A5011, 1:50; ABclonal) for 1 h. The cell nuclei were stained with DAPI (Beyotime) and cells were packaged on cover glasses with antifade mounting medium. Imaging was performed either with a Leica Tcssp8 laser scanning confocal microscope (Leica, Wetzlar, Germany) or a Leica DM4B positive fluorescence microscope (Leica).

### Statistical analysis

GraphPad Prism 5 software was used to prepare the figures and One-way analysis of variance (ANOVA) was used for comparison between multiple groups. *P*<0.05 is considered to be statistically different.

## Results

### Mutations at CD82 palmitoylation sites Cys5+Cys74 can promote EGFR internalization

To identify the effects of different mutations at CD82 palmitoylation sites on EGFR and c-Met, CD82 palmitoylation mutation plasmids were constructed and verified by sequencing ( Supplementary Figure S1B–H). After transfection, the cytoplasmic and membrane proteins of breast cancer MDA-MB-231 cells, with mutations at different palmitoylation sites of CD82, were separated and extracted. Western blot analysis results showed that when CD82 palmitoylation sites Cys5+Cys74 were mutated, the expression of CD82 and EGFR in the cytoplasm was increased. However, the expression of c-Met in the cytoplasm did not differ significantly between different CD82 palmitoylation site mutation groups, as shown in Figure 1B. Conversely, combined mutations at CD82 palmitoylation sites Cys5+Cys74 led to a decrease in the expressions of CD82 and EGFR on the cell membrane. There was still no significant difference in the expression of c-Met on the membrane among different mutation groups, as shown in Figure 1C.

The above results confirmed that the mutations at different palmitoylation sites of CD82 have no significant effect on the expression and location of c-Met, and therefore, the subsequent experiments will focus on the expression and metabolism of CD82 and EGFR. With double-point mutations at Cys5+Cys83, CD82 was expressed the most in terms of total protein, which was significantly different from the wild-type (WT) group and the normal control (N) group. Meanwhile, in the groups of single-point mutation at Cys83, double-point mutations at Cys74+Cys83, and triple-point mutations at Cys5+Cys74+Cys83, as well as in the WT group, CD82 expression (total protein) was slightly lower than that in the group of double-point mutations at Cys5+Cys83, which was significantly different from that in the normal control group. Furthermore, our results suggested that in the group of double-point mutations at Cys5+Cys74, CD82 expression did not differ from that in the normal control group in terms of total protein, as shown in Figure 1D. However, the expression level of EGFR (total protein) did not differ significantly among the groups, as shown in Figure 1E. Real-time PCR results showed that mRNA expression of CD82 was found in each CD82 palmitoylation site mutation group, as well as in the WT group, and all the CD82 palmitoylation mutation groups and WT group have higher CD82 expression than the control normal control group, as shown in Figure 1F.

### The internalized EGFR can bind to CD82 and co-localize in the recycling endosome

**Figure 2:**
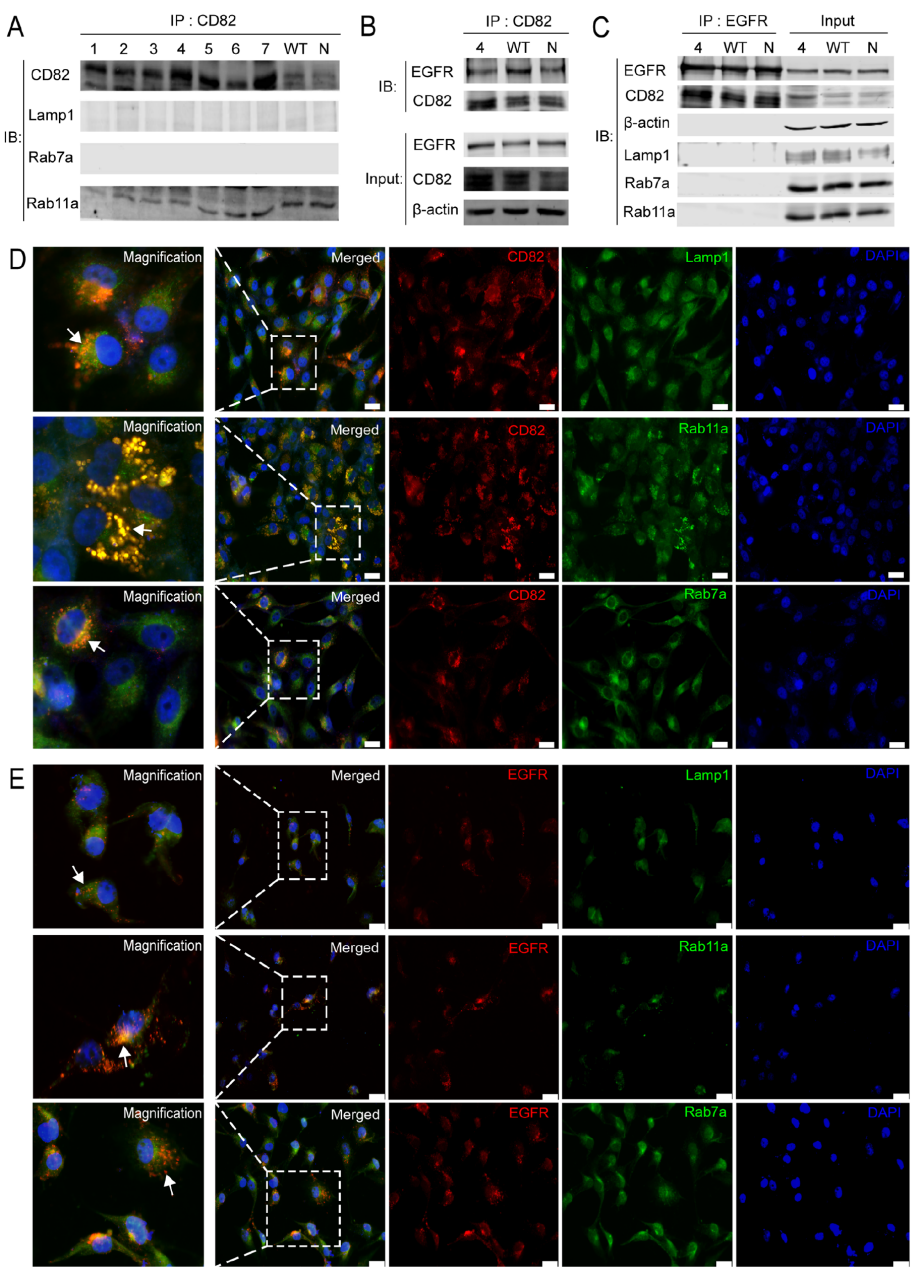
Figure2 **The interaction and co-localization of CD82 and EGFR**(A) After the mutations at different palmitoylation sites of CD82, the immunoblotting results showed the physical interaction relationship between CD82 and Lamp1, Rab7a and Rab11a. (B) CD82 could directly interact with EGFR in the CD82 palmitoylation Cys5+Cys74 sites combined mutation group, WT group and normal control group, and protein expression was also found in the input group. (C) EGFR in the CD82 palmitoylation Cys5+Cys74 sites combined mutation group, WT group and normal control group, the IP results showed that EGFR can directly bind to CD82, but not to Lamp1, Rab7a or Rab11a. In the input group, CD82 palmitoylationCys5+Cys74 sites combined mutation group, WT group and normal control group all had protein expression. (D) and (E) When the CD82 palmitoylation sites Cys5+Cys74 were mutated, co-localization of CD82 and EGFR separately with Lamp1, Rab11a and Rab7a. Scale bar=25 μm.

After the mutations of the different palmitoylation sites of CD82, CD82 was immunoprecipitated with Lamp1 (lysosomal marker), Rab7a (late endosome marker), and Rab11a (recycling endosome marker) in each mutation group. The results showed that CD82 can directly bind with Rab11a, and the two have a direct interaction relationship, as shown in Figure 2A. However, CD82 could not directly bind with Lamp1 or Rab7a. To further explore the molecular mechanism of the enhanced internalization of EGFR into the cytoplasm when the CD82 palmitoylation sites Cys5+Cys74 were mutated, the interaction between EGFR and CD82 was detected by immunoprecipitation assay, and the results showed that CD82 could directly bind to EGFR, as shown in Figure 2B. Similarly, the immunoblotting results showed that EGFR could directly bind to CD82, however, EGFR could not directly bind to Lamp1, Rab7a, or Rab11a, as shown in Figure 2C. Thus, we used immunofluorescence method to detect the co-localization relationship of CD82 with the three endosomal markers. We further explored the co-localization relationship of EGFR with the three endosomal markers. The immunofluorescence results showed that the co-localization of CD82 with Rab11a was the most evident, as shown in Figure 2D. The degree of co-localization of EGFR with Rab11a was also higher than that with Lamp1 and Rab7a, as shown in Figure 2E.

### Mutations in CD82 palmitoylation sites Cys5+Cys74 can promote EGFR metabolism in the recycling endosome pathway

**Figure 3:**
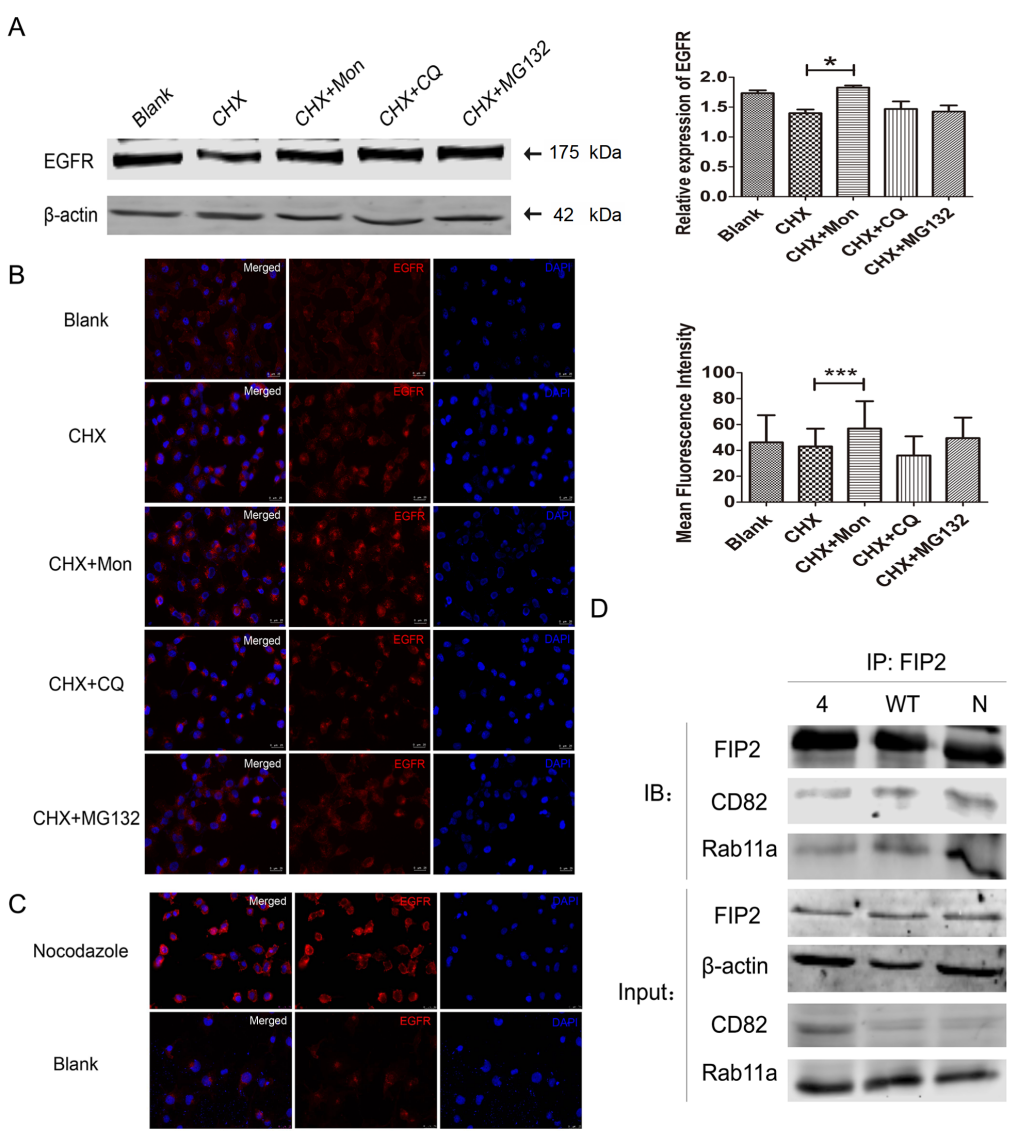
Figure3 **EGFR was metabolized through the recycling endosome pathway by forming a complex**(A) Western blot analysis and (B) immunofluorescence assay were used to detect the expression of EGFR in the total protein with different inhibitors after CD82 palmitoylation sites Cys5+Cys74 were mutated. Scale bar=25 μm. (C) After the combined mutation of the CD82 palmitoylation sites Cys5+Cys74, the tubulin inhibitor nocodazole (66 μM, 4 min) was added to detect the expression of EGFR. The upper row is the nocodazole group, the bottom row is the control group. Scale bar=25 μm. (D) FIP2 in the CD82 palmitoylation sites Cys5+Cys74 combined mutation group, WT group and normal control group, the IP results showed that FIP2 can directly bind to Rab11a and CD82. In the input group, the CD82 palmitoylation sites Cys5+Cys74 combined mutation group, WT group and normal control group all had protein expression.

Cycloheximide (CHX) is a protein translation inhibitor which inhibits the process of protein translation, thereby blocking protein expression. Monensin (Mon) is a protein transport inhibitor that suspends the transport process from endosomes to cell membranes and also inhibits recycling endosomes. In addition, chloroquine (CQ) is a lysosomal inhibitor, and MG132 is a proteasome inhibitor. Western blot analysis results revealed that when treated with 1 mM CHX for 8 h, EGFR expression was decreased, and when 10 μM Mon was added, EGFR expression was resumed and increased ( Figure 3A). The cells were treated with the same concentrations of drugs, and immunofluorescence experiments were performed. When the cells were treated with 10 μM Mon, the average fluorescence intensity of EGFR was significantly increased, as shown in Figure 3B. Taken together, EGFR is metabolized through the circulating body.

In this experiment, nocodazole was selected to inhibit the aggregation of microtubules. When the CD82 palmitoylation sites Cys5+Cys74 were mutated, immunofluorescence results clearly showed that compared with the control group, the nocodazole-treated (66 μM, 4 min) group had a decreased expression of EGFR in the cytoplasm and an increased expression of EGFR on the cell membrane, as shown in Figure 3C. These results suggested that the internalization process of EGFR requires the action of tubulin.

FIP2 is a member of the Rab11 interaction family. In the double-point mutation group at Cys5+Cys74, WT group and normal control group, the interaction between FIP2 and Rab11a was detected by immunoprecipitation assay. Western blot analysis results showed that FIP2 could directly bind to Rab11a, and simultaneously FIP2 could also directly bind to CD82. In the input control group, CD82, FIP2 and Rab11a were all expressed with mutations in the CD82 palmitoylation sites Cys5+Cys74, as shown in Figure 3D.

## Discussion

Tetraspanin CD82 can regulate the occurrence, development, and metastasis of most tumors, thereby inhibiting tumor metastasis [ 18– 20]. After palmitoylation modification, the tetraspanin protein located in the cytoplasm can anchor on the cell membrane, and then promote the formation of tetraspanin web, signal transport process and stably maintain normal physiological functions [ 21– 24]. CD82 can weaken the EGF/EGFR induction signal and inhibit tumor metastasis, but the mechanism is still unclear [ 25– 28]. The effect of CD82 palmitoylation site mutation on the expression, location, and metabolism of EGFR and c-Met in breast cancer cells remains unclear so far. In this study, by constructing mutants with different CD82 palmitoylation site mutations, we explored the influence of palmitoylation site mutations on the expression, distribution, and metabolic pathways of EGFR and c-Met.

When the Cys5+Cys74 in CD82 were mutated, the internalization ability of EGFR was strengthened. However, more EGFR could not be stably expressed on the cell membrane, and it was transferred from the cell membrane to the cytoplasm. We further observed that, in total protein, among different CD82 palmitoylation site mutation groups, there was no significant difference in EGFR expression. We confirmed that CD82 palmitoylation mutations at different sites cannot change the total expression of EGFR, but change the distribution of EGFR in cytoplasm and cytomembrane. CD82 palmitoylation site mutations could not affect the expression or localization of c-Met, indicating that CD82 regulation of c-Met in tumor cells requires the participation of other post-translational modifications or the assistance of other signaling molecules, which needs to be studied in the future. For CD82 itself, palmitoylation site mutations at Cys5+Cys74 also changes the distribution of CD82 in cytoplasm and cytomembrane.

**Figure 4:**
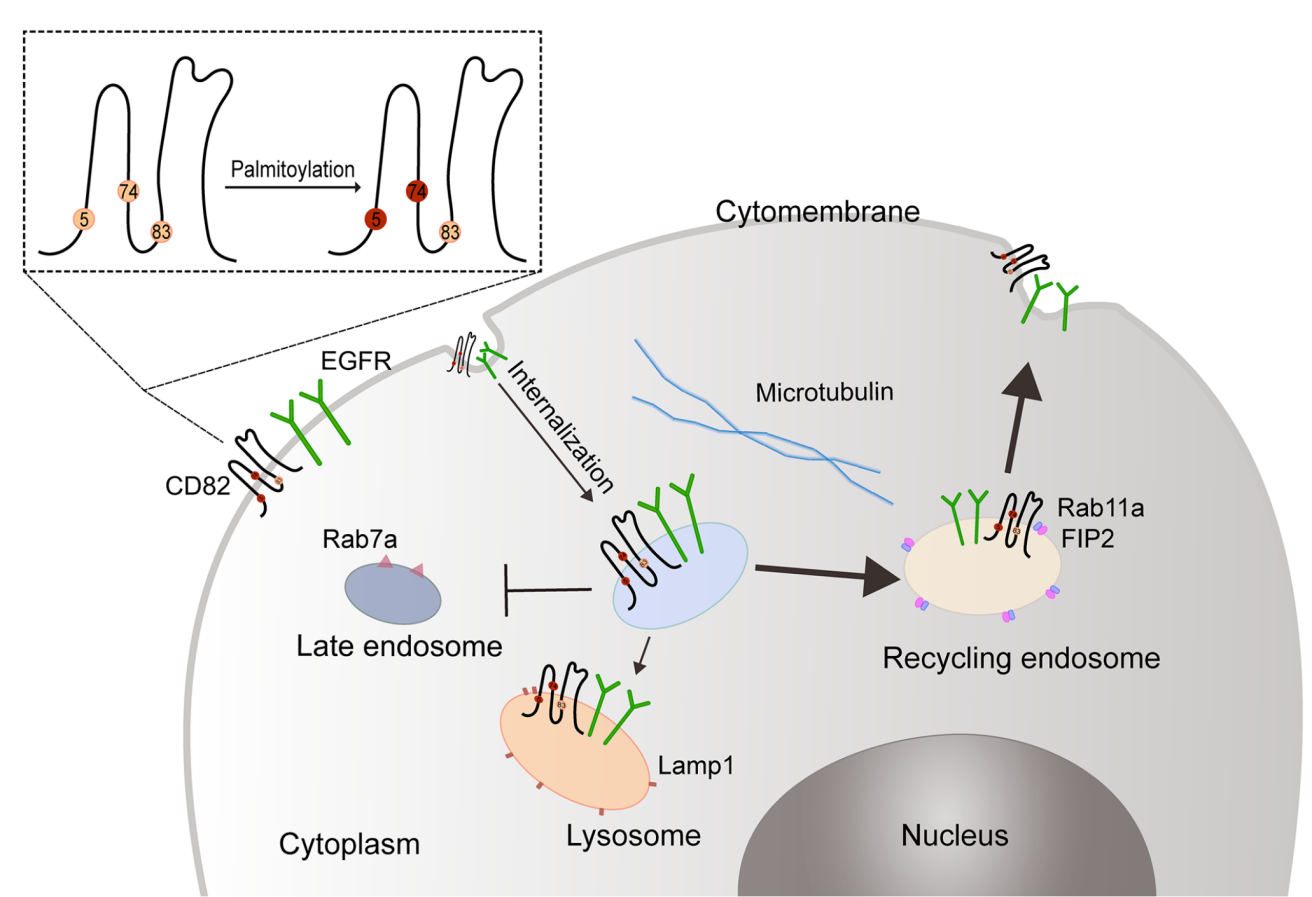
Figure4 **A mechanistic diagram of the effect of the tetraspanin CD82 palmitoylation site mutations on the expression and localization of EGFR**When the CD82 palmitoylation sites Cys5+Cys74 of are mutated, the internalization ability of EGFR is enhanced. After internalization, most EGFR and CD82 enter the recycling endosome in a direct binding manner. This process requires the assistance of tubulin. CD82 can directly bind with Rab11a and FIP2 to form an EGFR/CD82/Rab11a/FIP2 complex, which is metabolized by the recycling endosome pathway to re-express EGFR and CD82 on the cell membrane surface. The remaining small part of the EGFR/CD82 complex is degraded in the lysosome, and this metabolic process does not go through the late endosome pathway.

Therefore, the following hypothesis can be put forward. These internalized EGFR and CD82: (1) may be decomposed through certain metabolic pathways, such as lysosome pathway, (2) can circulate through the recycling endosome pathway, and (3) be processed through late endosome pathway ( Figure 4). Lamp1, Rab11a, and Rab7a are lysosome markers, recycling endosome markers, and late endosome markers, respectively [29]. With palmitoylation site mutations at Cys5+Cys74, most of the CD82 and EGFR with enhanced internalization ability are located in the recycling endosome. This implies that most CD82 and EGFR are metabolized through the recycling endosome pathway, and a small part is metabolized through the lysosome pathway.

Studies have demonstrated that CD82 can directly bind to EGFR and inhibit EGF-induced cell migration, and the process of weakening of the EGFR signal may be related to endocytosis [30]. In this study, when CD82 palmitoylation sites Cys5+Cys74 were mutated, the enhancement of EGFR internalization was also related to the direct binding of EGFR with CD82, and EGFR and CD82 were transferred into the cytoplasm in the form of direct binding. Endosomes can be transported along microtubules with the assistance of dynein [31]. In this study, to further verify the specific molecular mechanism of EGFR internalization into the cytoplasm after mutations at the CD82 palmitoylation sites Cys5+Cys74 and metabolization through the recycling endosome pathway, the tubulin inhibitor nocodazole was used to inhibit the aggregation of tubulin. When nocodazole was added, most EGFR was located on the cell membrane, and the internalization ability was weakened. Therefore, it can be inferred that after CD82 palmitoylation site mutations at Cys5+Cys74, EGFR is internalized through the binding with CD82, and this process also requires the assistance of tubulin.

To further verify that the CD82 palmitoylation site mutations at Cys5+Cys74 causes EGFR to be internalized and then metabolized through the recycling endosome pathway, monensin was selected as an inhibitor of the recycling endosome pathway. Simultaneously, cycloheximide was selected to inhibit EGFR production, and chloroquine was compared with MG132 as a control group [ 29, 32]. When monensin was added, the effect of cycloheximide on the reduction of EGFR expression was largely restored. This suggests that when the recycling endosome pathway is inhibited, the EGFR metabolism is blocked, which reversely ensures that EGFR is metabolized through the recycling endosome pathway.

Rab11a can be used as a marker protein of the recycling endosome [33]. FIP2 is one of the subfamily members of Rab11-FIPs and plays an important regulatory role in the process of molecular recycling of the cell surface [ 34– 37]. Rab11a can recruit myosin Vb and cytoplasmic dynein through the effectors, FIP2 and FIP3 [38]. With CD82 palmitoylation site mutations at Cys5+Cys74, FIP2 could directly bind to Rab11a and CD82. Therefore, it can be inferred that CD82, Rab11a, and FIP2 form a complex to assist the recovery of EGFR on the cell membrane. At the same time, CD82 is also expressed on the cell membrane again, as shown in Figure 4.

Nevertheless, in this study we only explored the metabolic pathways of EGFR after mutations at the CD82 palmitoylation sites Cys5+Cys74. The metabolic pathways in other mutants and the mechanism of CD82 internalization enhancement are still unclear. Whether the EGFR and CD82 located in the lysosome are completely degraded remains to be verified.

In summary, when the Cys5+Cys74 palmitoylation sites of CD82 are mutated, EGFR is transferred from the cell membrane to the cytoplasm under the CD82 mediation and located on the recycling endosome under the co-transportation of tubulin. Furthermore, by forming an EGFR/CD82/Rab11a/FIP2 complex, EGFR and CD82 are transported and recovered to the cell membrane for re-expression. Studies on the metabolic pathways and mechanisms of CD82 and tumor metastasis-related factors in breast cancer cells will help further understand the mechanism of breast cancer formation and metastasis and provide ideas for more precise targeted therapy.

## Supplementary Data

Supplementary data is available at *Acta Biochimica et Biophysica Sinica* online.

## Supporting information

Supplementary_data
