# Supplementary material for: CD82 palmitoylation site mutations at Cys5+Cys74 affect EGFR internalization and metabolism through recycling pathway: CD82 palmitoylation mutation can regulate the localization of EGFR
Source: Acta Biochim Biophys Sin (Shanghai). 2022 Feb 23;54(3):400–8. doi: 10.3724/abbs.2022011 (PMC9828285; doi:10.3724/abbs.2022011)

**Supplementary data**

**
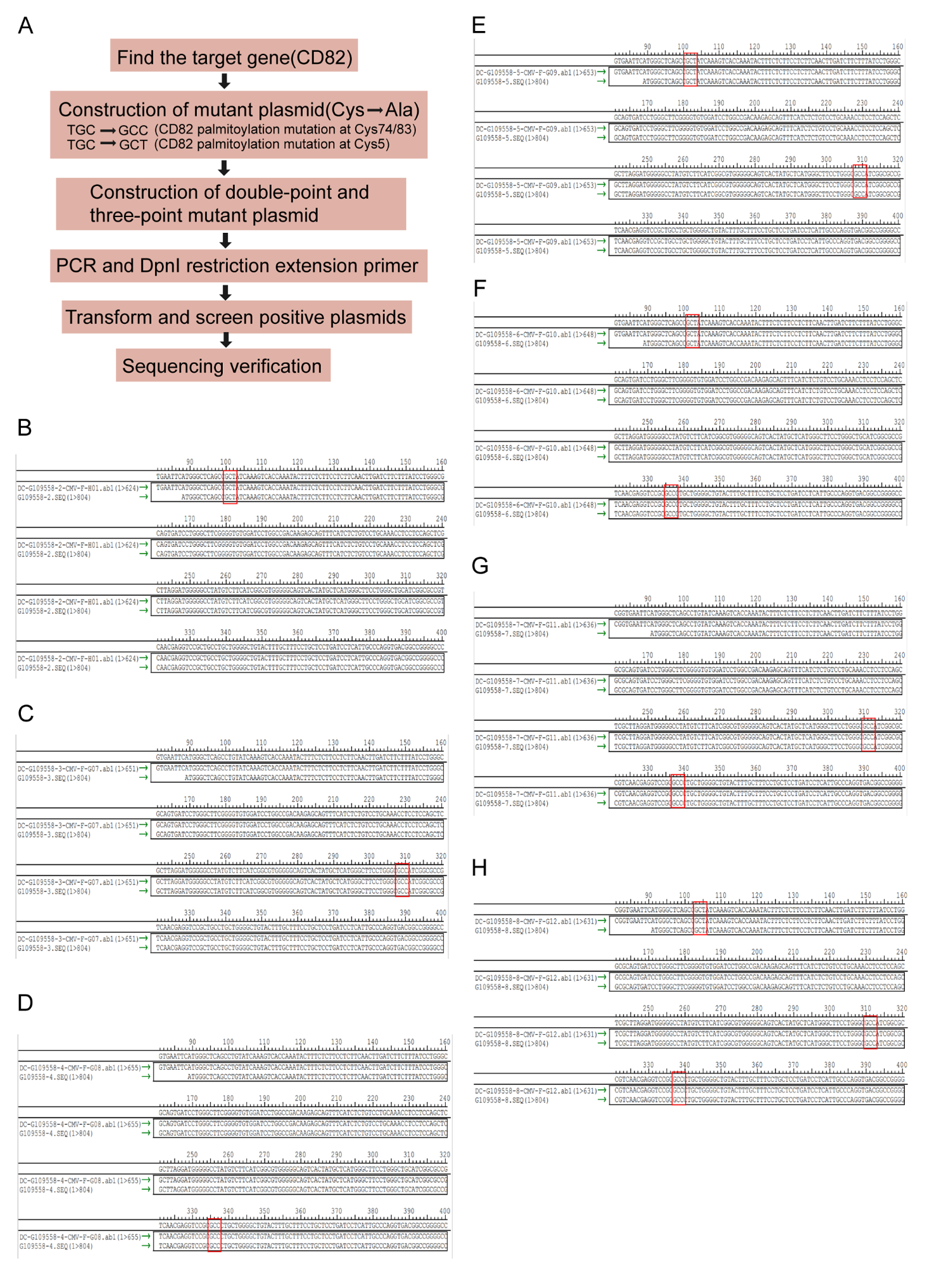
**

**Supplementary Figure S1. CD82 palmitoylation mutation plasmid construction and sequencing** (A) The flow chart of CD82 palmitoylation mutant plasmid construction. (B‒H) Sequencing results of CD82 palmitoylation mutation at Cys5 (B), Cys74 (C), Cys83 (D), Cys5+Cys74 (E), Cys5+Cys83 (F), Cys74+Cys83 (G), Cys5+Cys74+Cys83 (H).

**Supplementary Table S1. Primers used in CD82 palmitoylation mutation plasmid construction**


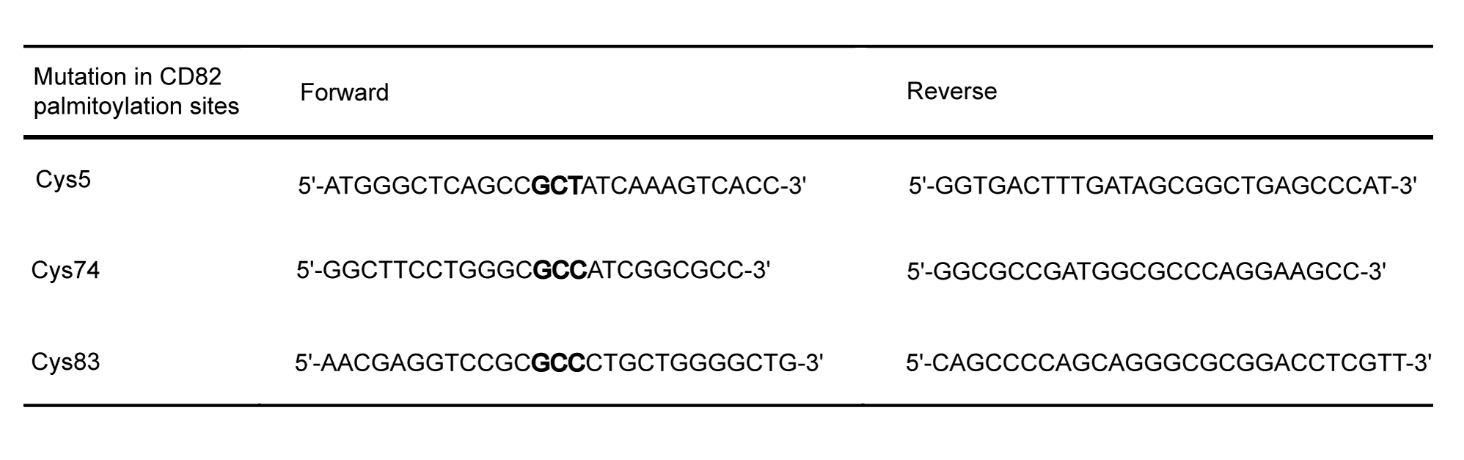

Supplement: Supplementary_data [file Supplementary_data.doc]
